# The physiological role of Motin family and its dysregulation in tumorigenesis

**DOI:** 10.1186/s12967-018-1466-y

**Published:** 2018-04-12

**Authors:** Tingting Huang, Yuhang Zhou, Jinglin Zhang, Alfred S. L. Cheng, Jun Yu, Ka Fai To, Wei Kang

**Affiliations:** 1 Department of Anatomical and Cellular Pathology, State Key Laboratory of Oncology in South China, Prince of Wales Hospital, The Chinese University of Hong Kong, Shatin, N.T, Hong Kong People’s Republic of China; 2 Institute of Digestive Disease, Partner State Key Laboratory of Digestive Disease, The Chinese University of Hong Kong, Shatin, N.T, Hong Kong People’s Republic of China; 3 Li Ka Shing Institute of Health Science, Sir Y.K. Pao Cancer Center, The Chinese University of Hong Kong, Shatin, N.T, Hong Kong People’s Republic of China; 4 Shenzhen Research Institute, The Chinese University of Hong Kong, Shenzhen, People’s Republic of China; 5 School of Biomedical Sciences, The Chinese University of Hong Kong, Hong Kong, People’s Republic of China; 6 Department of Medicine and Therapeutics, The Chinese University of Hong Kong, Hong Kong, People’s Republic of China

**Keywords:** AMOT, AMOTL1, AMOTL2, Hippo pathway, YAP1, Cancer

## Abstract

Members in Motin family, or Angiomotins (AMOTs), are adaptor proteins that localize in the membranous, cytoplasmic or nuclear fraction in a cell context-dependent manner. They control the bioprocesses such as migration, tight junction formation, cell polarity, and angiogenesis. Emerging evidences have demonstrated that AMOTs participate in cancer initiation and progression. Many of the previous studies have focused on the involvement of AMOTs in Hippo-YAP1 pathway. However, it has been controversial for years that AMOTs serve as either positive or negative growth regulators in different cancer types because of the various cellular origins. The molecular mechanisms of these opposite roles of AMOTs remain elusive. This review comprehensively summarized how AMOTs function physiologically and how their dysregulation promotes or inhibits tumorigenesis. Better understanding the functional roles of AMOTs in cancers may lead to an improvement of clinical interventions as well as development of novel therapeutic strategies for cancer patients.

## Background

**Fig. 1:**
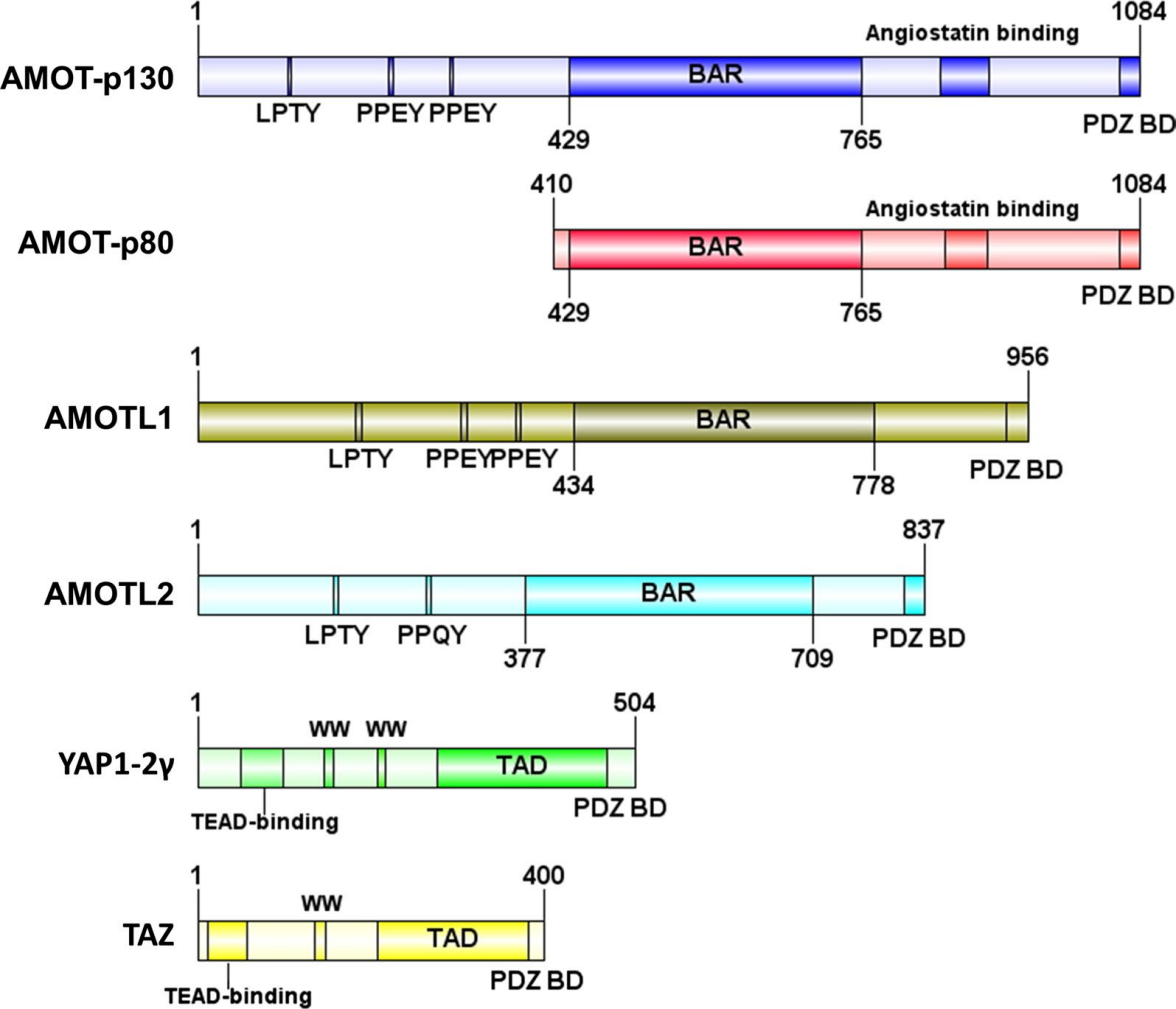
Schematic illustrations of the domain structures and motifs of the Motin protein family and YAP1-2γ/TAZ. Because of the alternative splicing, AMOT-p80 is an N-terminal truncated protein of AMOT-p130. The N-terminal domain of AMOT-p130 contains LPTY motif and two PPEY motifs, which are required for YAP1-binding. Except for the Angiostatin-binding domain, AMOTL1 and AMOTL2 share sequence identity with AMOT-p130. In addition, all members of Motin possess a Bin/Amphiphysin/Rvs (BAR) domain. Prominent regions of YAP1-2γ/TAZ include WW domain(s), TEAD transcription factor-binding domain, transcriptional activation domain (TAD) and post synaptic density protein (PSD95) binding domain (PDZ BD). WW domains are required by AMOTs binding

**Fig. 2:**
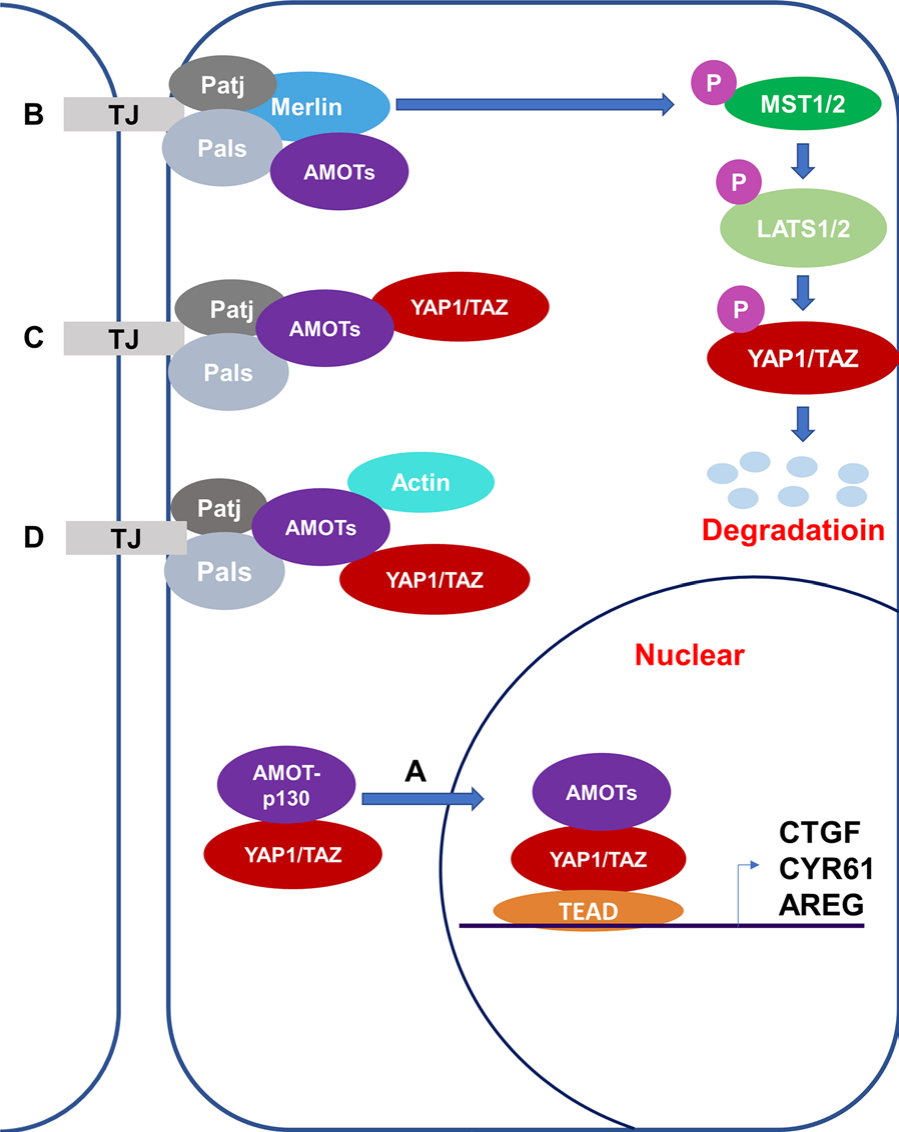
Schematic models of AMOTs interplayed with Hippo-YAP1 cascade. In this model, AMOTs mainly inhibits YAP1 by regulating its localization and promoting Hippo-mediated phosphorylation of YAP1. **a** AMOTs and YAP1 were co-translocated to the nuclear to promote the transcription of YAP1-target genes. **b** AMOTs bind with Merlin at the tight junctions and phosphorylate MST1/2 and LATS1/2. Phosphorylated LATS1/2 inactives YAP1 through phosphorylation, resulting in its degradation. **c** AMOTs physically interact with YAP1 to maintain it cytoplasmic retention. **d** Actin and YAP1 compete for binding with AMOTs in the tight junctions. TJ tight junction

Motin family, also known as AMOTs, consist of three members: Angiomotin (AMOT), Angiomotin-like 1 (AMOTL1) and Angiomotin-like 2 (AMOTL2). AMOT was first identified as an angiostatin binding protein that regulated endothelial cell migration by yeast two-hybrid screening [1]. AMOTL1 and AMOTL2 are two human protein sequences in Motin family, which are similar to AMOT [2]. These proteins exhibit significant sequence homology and share several structural characteristics [2]. A later study identified two isoforms of AMOT, -p80 and -p130 [2, 3]. AMOT-p130 arises from alternative splicing of the *AMOT* gene between exons 2 and 3 with an extended 409 amino acids at N-terminus. Through alternative splicing, *AMOT* gene also produces AMOT-p80 which lacks the N-terminal 409 amino acids [4]. AMOT-p130, AMOTL1, and AMOTL2 share the same N-terminus, which is composed of conserved glutamine-rich domains, LPTY motif and PPXY motifs (AMOT-p130: ^239^PPEY^242^ and ^284^PPEY^287^; AMOTL1: ^310^PPEY^313^ and ^367^PPEY^370^; AMOTL2: ^210^PPQY^213^). PPXY motifs specifically interact with YAP1/TAZ via the WW domain of YAP1/TAZ (Fig. 1) [5–7]. In addition, a GST pull-down assay demonstrated that deletion of a single LPTY motif abolished the binding with YAP1, whereas deletion of both PPEY motifs still retained some ability to interact to YAP1. These results suggested that the LPTY is also critical for YAP1 interaction [8]. LPTY motifs were well reserved in AMOTL1 and AMOTL2 [8]. In the C-terminal region, they (AMOT-p80, AMOT-p130, AMOTL1, and AMOTL2) all compose of the conversed Bin/Amphiphysin/Rvs (BAR) domain and the C-terminal PDZ-binding domain [9]. Although there is high similarity among AMOT family members, their various functions are not fully understood. Molecular diversity within the AMOTs have also been revealed [10]. Functionally, AMOTs is involved in Hippo signaling pathway through interacting with multiple core proteins on this pathway, such as Merlin, MST1/2, and YAP1 (Fig. 2). Recent studies have demonstrated several features of AMOTs in numerous pathways, which are directly linked to the initiation and progression of cancers. Particularly, these members function oppositely as oncogenes or tumor suppressive genes depending on different cellular context. For example, most of studies suggested oncogenic functions of AMOT-p80, such as in hemangioendothelioma, head and neck squamous cell carcinoma, and prostate cancer. While for AMOT-p130, it could exert both its oncogenic functions and tumor suppressive functions according to literatures. Currently, AMOTL1 was mainly reported as oncogene in breast cancer and cervical cancer. AMOTL2 is also a promoter of breast cancer progression while it suppressed glioblastoma carcinogenesis (Table 1). However, specific molecular mechanisms behind this situation are not clearly demonstrated. In this review, we will introduce the physiological role of the Motin family members, their controversial indications in cancer, and the mechanisms toward Hippo or non-Hippo pathways.

**Table 1:** Functional role of AMOT, AMOTL1, and AMOTL2 in different cancer types

	Functions	Cancer type	Mechanism	References
AMOT	Oncogene	Unknown	A DNA vaccine targeting AMOT inhibits angiogenesis and suppresses tumor growth. Therapeutic antibodies targeting AMOT inhibit angiogenesis in vivo. A vaccine targeting AMOT hampers tumor growth. sCD146 binds to Amot to stimulate a proangiogenic response. Tankyrase inhibitors antagonizes stabilize AMOT and result in constitutive activates of TEAD-dependent transcription and proliferation of human tumor cells	[29, 62, 79–81]
		Breast cancer	AMOT is up-regulated and its expression links to the aggressive nature of breast cancer. It promotes breast cancer cell proliferation and invasion. AMOT increases the expression of YAP1 in the nucleoprotein. miR-205 inhibits the proliferation and invasion of breast cancer by regulating AMOT expression	[71, 78, 89]
		Sinonasal inverted papilloma	AMOT is over-expressed. It associates with progression and growth via promoting angiogenesis in sinonasal inverted papilloma	[83]
		Osteosarcoma	lncRNA SNHG12 promotes cell proliferation and migration by activating AMOT gene expression. Also, miR-497 inhibits cell proliferation, migration, and invasion by targeting AMOT	[87, 88]
		Renal cell carcinoma	AMOT promotes cell proliferation by retaining the nuclear YAP1	[98]
		Colon cancer	AMOT promotes the malignant potential of colon cancer cells by activating the YAP1-ERK/PI3 K-AKT signaling pathway	[86]
	Tumor suppressor	Unknown	Form a TJ-associated protein complex with Merlin, Patj, and Pals1. AMOT inhibits MAPK signaling. AMOT inhibits YAP1 oncoprotein and restricts the activity of YAP1/TAZ. AMOT activates LATS2. Tankyrase inhibitors stabilize AMOT family proteins and suppress YAP1 oncogenic functions. Deubiquitylation of AMOT at lysine 496 by USP9x resulting in stabilization of AMOT and lower YAP1/TAZ activity	[6, 48, 55, 61, 65, 98]
		Lung cancer	AMOT decreases lung cancer progression by sequestering oncogenic YAP1/TAZ and decreasing Cyr61 expression. Tankyrase inhibitor sensitizes lung cancer cells to endothelial growth factor receptor inhibition via stabilizing AMOT and inhibiting YAP1 signaling	[59, 60]
AMOT-p80	Oncogene	Hemangioendothelioma	AMOT-p80 promotes angiogenesis by stimulating invasion and stabilizing established tubes	[77]
		Head and neck squamous cell carcinoma	High expression AMOT-p80 promotes cell proliferation and migration	[84]
		Prostate cancer	AMOT-p80 is a novel component of cadherin-11/β-catenin/p120 complex and promotes cell migration	[85]
AMOT-p130	Oncogene	Unknown	AMOT-p130 acts as a YAP1 cofactor, preventing YAP1 phosphorylation and augmenting its activity	[75]
	Tumor suppressor	Unknown	AMOT-p130 and AIP4 cooperatively reduces YAP1 and cell growth. AMOT-p130 selectively induced YAP1 phosphorylation and reduced transcription of connective tissue growth factor in an AIP4-dependent manner. AMOT-p130 decreased the growth of MDA-MB-468 breast cancer cells. AMOT-p130 (S175A)-expressing cells formed enlarged and poorly differentiated acini	[64, 66]
AMOTL1	Oncogene	Breast cancer	AMOTL1 marginally expressed higher levels in tumor than normal tissues. AMOTL1 promotes breast cancer progression and is antagonized by Merlin. AMOTL1 is an essential effector of the N-cadherin mediated endothelial/pericyte junctional complex	[39, 78, 99]
		Cervical cancer	MiR-124 represses vasculogenic mimicry and cell motility by targeting AMOTL1	[100]
	Tumor suppressor	Unknown	AMOTL1 activates LATS2, inhibits YAP1, and restricts the activity of TAZ and YAP1. It inhibits YAP1′s nuclear translocation and pro-apoptotic function	[51, 52, 55, 65, 69]
AMOTL2	Oncogene	Unknown	AMOTL2 promotes cell migration and proliferation of angiogenic endothelial cells. It positively regulates MAPK/ERK activation	[40]
		Breast cancer	AMOTL2 marginally expressed higher in tumors. It disrupts apical–basal cell polarity and promotes tumor invasion	[78, 90]
	Tumor suppressor	Unknown	AMOTL2 regulates YAP1 cytoplasm-to-nucleus translocation. AMOTL2 inhibits epithelial-mesenchymal transition. LATS2, AMOTL2, and YAP1 all localize to TJs, trigger LATS2 activation and growth inhibition in response to increased cell density. AMOTL2 mono-ubiquitination is required for YAP1 inhibition	[54, 65, 69]
		Glioblastoma	mTORC2/AMOTL2/YAP1 signaling cascade promotes glioblastoma growth and invasive characteristics. AMOTL2 upregulation inhibited YAP1-induced transcription, foci formation, growth, and metastatic properties both in vitro and in vivo	[76]

## The physiological roles of AMOTs

AMOTs are differently expressed across different tissues and they show variable spatiotemporal and expressional patterns. The highest expression of AMOT was reported to be in testis, followed by brain and thyroid. AMOTL1 is also highly expressed in skeletal muscle and the lowest levels are found in blood [11]. As for AMOTL2, breast tissue exhibits the highest expression among all the tissues [11]. According to previous researches, endogenous AMOTs expressions have been evaluated in many cell lines, such as in endothelial, epithelial, neural cells and fibroblasts. AMOT can be detected in most of epithelial, endothelial, fibroblast and neural cell lines. Nevertheless, AMOTL2 could not be measured in most cell types of these four cell lines. For AMOTL1, it is presented in a large proportion of endothelial and fibroblast cells [11].

### Functional role of AMOTs in embryonic development

During the preimplantation stage, mouse embryos establish two cell lineages by the time of early blastocyst formation: the trophectoderm (TE) and the inner cell mass (ICM) [12]. AMOT, AMOTL1, and AMOTL2 behave variously in embryo development. In early mammalian embryo, AMOT is differentially expressed in the progenitors of the pluripotent ICM and the differentiated TE precursors [13, 14]. The appropriate formation of TE and ICM lineages is dependent on the differential activity of the Hippo-signaling pathway between the outer- and inner-cell populations [15]. The inner cells show high levels of Hippo pathway while outer cells of the embryo show low Hippo pathway [13, 14]. These different activities of Hippo pathway was at least partly regulated by AMOTs. At the adherent junctions of the inner cells, AMOT recruits and activates LATS1/2 to promote Hippo pathway [13, 14]. In apical membrane of the outer cells, F-actin inhibits the ability of AMOT to activate Hippo signaling [13, 14]. AMOT phosphorylation disrupts AMOT—F-actin interaction and leads to reduced F-actin stress fibers and focal adhesions. Also, this phosphorylation was reported to suppress endothelial cell migration in vitro and angiogenesis in zebrafish embryos in vivo [16]. The localization of AMOT is regulated by Rho-associated kinase (Rock) to regulate appropriate apical-basolateral polarization in outer cells [17]. In AMOTL2 knockdown (KD) embryos, they exhibit a normal YAP1 distribution and Hippo pathway activation. However, with AMOTL2 KD in AMOT-null mutants, these embryos exhibit strong nuclear localization of YAP1 in the inner cells, suggesting that some Motin family proteins are essential for the activation of Hippo pathway in preimplantation embryos [18]. During the peri-implantation period, AMOT, AMOTL1, and AMOTL2 are differentially expressed in uterine cells [19]. There was evidence showing a transitional expression pattern of AMOT during the embryonic development: from the anterior visceral endoderm (AVE) to the visceral endoderm (VE) subsequently, and the latter was associated with extra-embryonic ectoderm. In subregions of VE, AMOT regulated morphogenetic movements that were required for embryo viability [20]. AMOT-null mutant embryos failed to exclude YAP1 from the nuclei of the inner cells until 32-cell stage [18]. The embryos survived until the postimplantation stages [18].

On the other hand, AMOTL2 is expressed maternally and in restricted cell types zygotically [21]. Silencing AMOTL2 expression impaired convergence and extension movement [21]. In mosaic embryos, cells with AMOTL2 knockdown failed to migrate properly [21]. AMOTL2 partially co-localized with RhoB- or EEA1- positive endosomes and tyrosine kinase c-Src, which in turn regulated the membrane architecture [21]. Thus, AMOTL2 is essential for cell movements in vertebrate embryos [21]. In zebrafish, AMOTL2 regulates embryonic development through inhibiting Wnt/β-catenin signaling [22]. It can interact with and trap β-catenin in the Rab11-positive recycling endosomes to reduce the amount of β-catenin in both the cytosol and nucleus [22].

### Roles of AMOTs in cell migration

AMOT was first identified to mediate angiostatin inhibition of endothelial migration and tube formation in vitro [1]. In zebrafish, knocking down AMOT was shown to decrease the number of filopodia of endothelial tip cells [23]. AMOT is also required for endothelial polarization during migration in that AMOT regulates Rac1 activity in endothelial and epithelial cells [23]. When transgenic mice expressed C-terminal deletion mutant AMOT through endothelial cell-specific receptors tyrosine kinase (TIE) promoter, migration of endothelial cells into the neuroectoderm and intersomitic regions was inhibited [24]. AMOT also interacts with tissue factor pathway inhibitor-1 (TFPI-1), which leads to YAP1 activation and further increases the genes expression relating to proliferation and migration [25]. In Madin–Darby canine kidney (MDCK) cells, overexpression of AMOT induces relocalization of polarity proteins and loss of transepithelial electrical resistance [26]. And the interaction between AMOT and Rich1 (RhoGAP interacting with Cdc42-interacting protein four homologues protein 1) is necessary for maintaining TJ integrity [26]. MUPP1, which has an MRE domain and 13 PDZ domains, is located at TJs. The interaction between MUPP1 and AMOT has been identified in epithelial cells by yeast two-hybrid screening [27]. Patj is a close relative of MUPP1. Possessing a similar structure, Patj interacts with all AMOT family members to regulate the formation of TJ and epithelial polarity [27]. Pdlim2, a member of actin-associated LIM proteins subfamily, is expressed exclusively by podocytes in kidney. AMOTL1 interacts with Pdlim2 and governs the dynamics of the actin cytoskeleton in foot processes [28].

### Roles of AMOTs in angiogenesis

Seventy five percentage of AMOT knockout mice died between embryonic day E11 and E11.5 [23]. Most of them exhibited severe vascular insufficiency in the intersomitic region and dilated vessels in the brain [23]. In addition, anti-AMOT antibody significantly inhibits endothelial migration and decreases the number of endothelial filopodia of tip cells during retinal angiogenesis [29]. Moreover, AMOT is a pivotal adaptor protein in the intersection between trafficking, cell junctions and cell migration, which plays a role in directional migration and angiogenesis [30]. Pals1-associated TJ protein (Patj), Lin Seven 1 (Pals1), and Par-3 are crucial for cell polarity. Together with Patj and synectin-binding GEF (Syx), AMOT forms a ternary complex. In vivo, AMOT plays an additive role with Syx in directing endothelial sprouts [31]. AMOT-Patj/multi-PDZ-domain protein 1 (Mupp1)-Syx govern the directional migration of capillaries in the embryo signaling through controlling RhoA GTPase activity to the leading front of migrating cells [32]. AMOT expression is up-regulated in dermal mesenchymal stem cells (DMSCs) in psoriasis, suggesting its involvement in the excessive angiogenesis and vasodilation [33].

AMOT distributes on cell surface and co-localizes with tight junction (zonula occludens) protein (ZO-1) in cell–cell contracts in endothelial cells in vitro and in angiogenic blood vessels of the postnatal mouse’s retina in vivo [3]. AMOT-p130 recruits ZO-1 to actin stress fibers, which is responsible for its localization to actin and tight junctions (TJs) [3]. Furthermore, AMOT-p130 coprecipitates with MAGI-1b, a component of endothelial TJs. Therefore, AMOT contributes to the assembly of endothelial cell–cell junctions [3]. AMOT-p80 and AMOT-p130 play coordinating roles in tube formation by affecting cell migration and cell shape respectively [4]. They were suggested to be differentially expressed in specific stages of mouse retina vascularization [34]. During retinal angiogenesis in vivo, AMOT-p80 was found to be expressed in the migratory phase [34]. In contrast, AMOT-p130 was detected during the period of blood vessel stabilization and maturation [34]. They have distinct functions, AMOT-p80 stimulated endothelial cell migration and angiogenesis while AMOT-p130 stabilized and matured vessels [34]. The rate of AMOT-p80 and AMOT-p130 expression served as an indicator for migration or stabilization of endothelial cells: increased AMOT-p80/-p130 ratio directly reflected the enhanced angiogenic ability of skeletal muscle in response to exercise training [35].

Similar to AMOT, AMOTL1 was also indicated to play a role in endothelial migration and TJ formation in vitro [36]. AMOTL1 mainly affected the stability of cell–cell junctions in stalk cells during sprouting angiogenesis in vivo [36]. It increased the velocity of migration and decreased the persistence of migration directionality. AMOTL1 interacted with AMOT-p80 to form a complex and induced the remodeling of actin cytoskeleton [37]. Motin family has also involved in controlling stability and permeability of endothelial cell junction. HECW2 (HECT, C2 and WW domain containing E3 ubiquitin protein ligase 2), the endothelial cell ubiquitin E3 ligase, physically interacts with AMOTL1 and enhances its stability [38]. In normal retinal, AMOTL1 is essential for normal establishment of vascular networks in the post-natal mouse retina. It forms a complex with N-cadherin to mediate endothelial junctional complex [39].

AMOTL2, accordingly, was detected in blood vessel cells, suggesting its critical roles in regulating multiple behaviors of endothelial cells during angiogenesis. In zebrafish transgenic embryos, knockdown of AMOTL2 impaired the intersegmental vessel growth and suppressed proliferation of endothelial cells [40]. AMOTL2 knockdown also inhibited cell proliferation and migration and disrupted cell polarity of cultured human umbilical vein endothelial cells [40]. It was required for MAPK/ERK activation during angiogenesis [40]. AMOTL2 also participates in aortic vessel lumen expansion through linking VE-cadherin to contractile actin fibers, which has been verified in zebrafish, mouse, and endothelial cell culture systems [41].

## The role of AMOTs in Hippo-YAP1 pathway

**Fig. 3:**
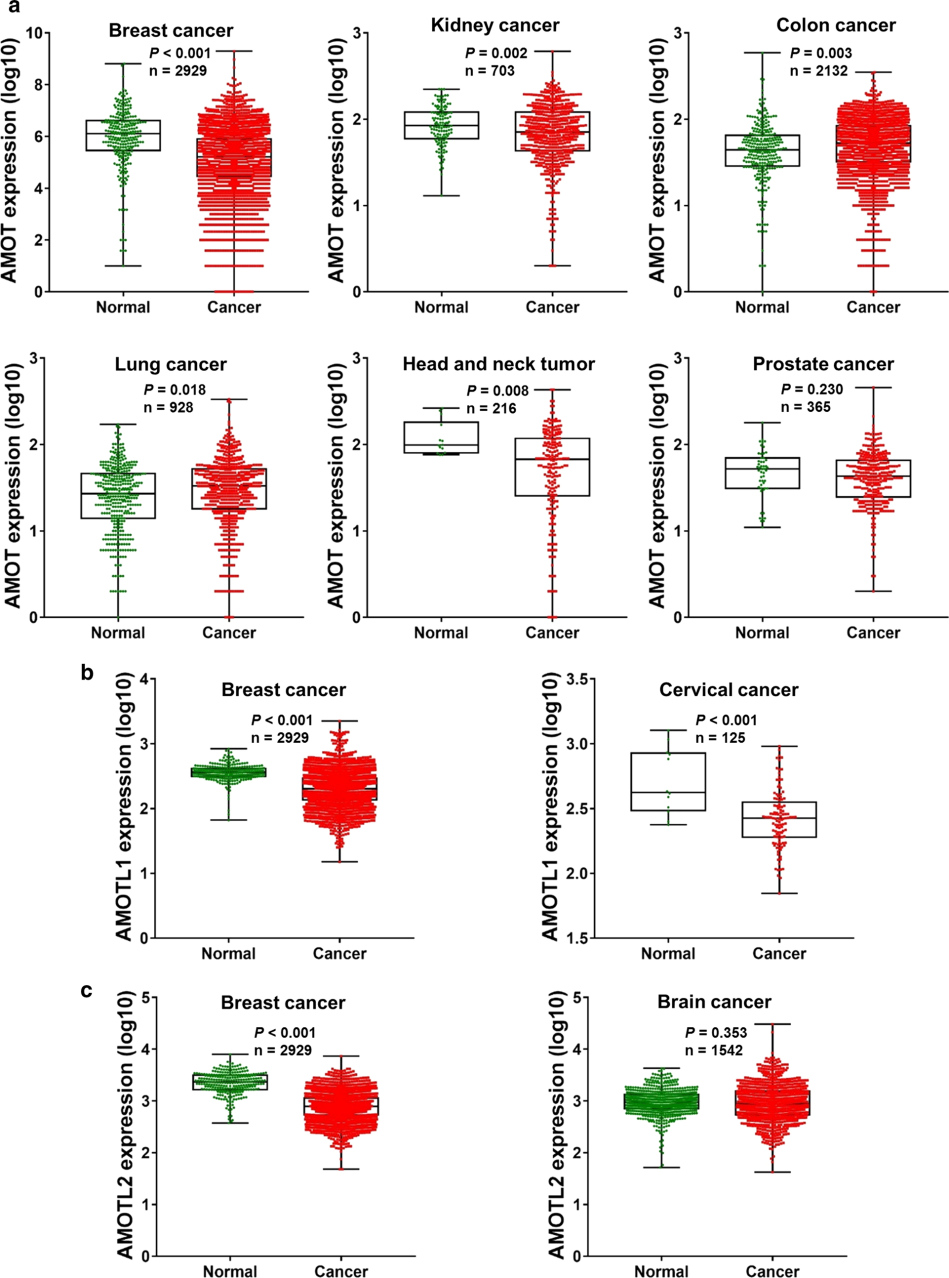
Expression pattern of Motin family members in various cancer types which have been reported previously. **a** According to GENT database, there is discrepancy of expression regarding to AMOT in different tumors. When compared to correspondingly normal control, its expression level is significantly lower in breast cancer (*P* < 0.001), kidney cancer (*P* = 0.002), as well as head and neck tumor (*P* = 0.008); however, AMOT is overexpressed in colon cancer (*P* = 0.003) and lung cancer (*P* = 0.018). The difference of AMOT expression between prostate cancer and the corresponding normal is insignificant (*P* = 0.230). **b** AMOTL1 exhibits downregulation in both breast cancer (*P* < 0.001) and cervical cancer (*P* < 0.001). **c** Expression of AMOTL2 is only down-regulated in breast cancer (*P* < 0.001), rather than brain cancer (*P* = 0.353)

AMOTs have been reported in kinds of cancer types (Fig. 3 and Table 1). According to GENT database, AMOT expression showed up-regulated in colon cancer and lung cancer while it is down-regulated in breast cancer, kidney cancer, head and neck tumor when comparing to correspondingly normal control tissues (Fig. 3a). Decreased AMOTL1 expression was observed in breast cancer and cervical cancer (Fig. 3b). Interestingly, suppressed and over-expressed AMOTL2 was verified in breast cancer and brain cancer respectively (Fig. 3c). Similar with their different expressional patterns, functional roles of AMOTs are distinct in various cancer types. For example, AMOT, AMOTL1, as well as AMOTL2 exert both oncogene or tumor suppressive gene in different cancer types (Table 1). In this review, we mainly focused on the functions of AMOTs in Hippo-YAP1 pathway. Meanwhile, different mechanisms were summarized based on their involvement in Hippo-YAP1 signaling and other pathways.

### Functional roles of AMOTs in Hippo-YAP1 pathway

Role of AMOTs in Hippo-YAP1 pathway is not clearly proven, even still highly controversial. Some laboratories have shown they inhibit cell proliferation, where others demonstrated the promotion of cell proliferation or tumor growth. Hippo pathway functions as an organ size controller, mainly through its ability to regulate cell proliferation and apoptosis [42, 43]. The core kinase cascade in mammals consists of MST1/2 kinases, WW45, LATS1/2 and Mob1. MST1/2 interacts with WW45 and phosphorylates Mob1 and LATS1/2, resulting in their activation [44]. Activated LATS1/2 phosphorylates YAP1/TAZ. YAP1 and TAZ are the nuclear effectors of Hippo pathway and they belong to the paralogous multifunctional co-activators [45]. AMOTs correlate with YAP1 through the binging of their PPXY and LPXY motifs with the WW domains of YAP1 and TAZ [5–7]. YAP1 is a transcriptional co-activator and a key component in Hippo pathway. Therefore, Motin family proteins have been also identified as novel components in Hippo-YAP1 pathway [6, 46].

Merlin functions upstream of the core Hippo pathway kinases LATS1/2 and MST1/2. An AMOT-dependent complex comprised of AMOT, YAP and Merlin has been described [47]. Merlin is a kind of TJ associated protein and interacts directly with AMOT through their mutual coiled-coil domains. In epithelial cells, AMOT retains Merlin at mature TJs. In addition, Merlin functions through AMOT and Rich1 to inhibit Rac1 and Ras-MAPK signaling. Interaction of AMOT and Merlin regulates Rac signaling via Cdc42/Rac1 GAP and Rich1. Therefore, AMOT is considered as a potential player in Merlin-related cancers [6, 48]. An elongated α-helix-rich region of AMOT (residues 404–728 of AMOT-p130) robustly bound to the entire C-terminal part of Merlin to form a stable complex [49]. This interaction releases the auto-inhibition and promotes Merlin’s binding to LATS1/2 to activate Hippo pathway [49].

AMOT was first identified as a partner of YAP1 via large screening of multi-protein complexes that were assembled on de-ubiquitinating enzymes [50]. In MDCK cells, AMOT inhibits YAP1-induced transformation and loss of cell contact inhibition. Knockdown AMOTL2 induces MDCK cell transformation in a YAP1/TAZ dependent manner [6]. AMOTL1, YAP1 and ZO-2 form a tripartite complex to regulate the function of YAP1 in HEK293 cells: AMOTL1 inhibits proapoptotic function of YAP1, while ZO-2 enhance it [51]. AMOTL1 was also reported to be stabilized by being directly phosphorylated by AMPK, which contributing to YAP1 inhibition [52]. On the other hand, AMOTL2 interacts with YAP1 and the Wnt/β-catenin effector Lef1 to control tissue size [53]. Moreover, mono-ubiquitination in turn mediates the function of AMOTL2 itself [54]. Moreover, Hippo-refractory-TAZ mutant (S89A) was reported to be negatively regulated by AMOT and AMOTL1. These results suggest a mechanism of Hippo pathway-independent restriction of TAZ and YAP1 by AMOTs, indicating that the function of AMOTs compensate for the absence of LATS1/2 kinases [55]. As for mammalian cells, AMOT could also regulate YAP1 activity through competitively interacting with endosomal integral membrane protein endotubin (EDTB) [56]. Overexpression of AMOT and AMOTL1 leads to the cytoplasmic retention of TAZ and downregulation of CTGF and Cyr61 expression. Cytoplasmic YAP1 recruits c-Abl to protect AMOTL1 against Nedd4.2-mediated degradation. Therefore, cytoplasmic YAP1 involves in the protecting of AMOTL1 [57].

### Tumor suppressive functions of AMOTs

Tumor suppressive functions of AMOTs have been identified in different cancer types. Overexpression of YAP1 impairs the nuclear retention of SRSF1 (serine/arginine-rich splicing factor 1) and itself via AMOT [58]. Therefore, AMOT mediates translocation of SRSF1 through YAP1 in liver cancer cells [58]. In clinical lung cancer specimens, AMOT expression was found to be significantly decreased, which indicated its tumor suppressive role. In vivo, AMOT knockdown increased the growth and spread of Lewis lung carcinoma [59]. Besides, tankyrase inhibitor sensitized cancer cells to endothelial growth factor receptor (EGFR) inhibition through stabilizing AMOT and inhibiting YAP1 signaling in lung cancer cells [60]. Tankyrases inhibitors stabilize AMOT family proteins, disabling the oncogenic function of YAP1 [61, 62]. Through controlling the stability of the AMOT family proteins, E3 ligase ITCH, and the LATS kinases, DUB3 has the potential to act as a tumor suppressor by limiting YAP/TAZ activity [63]. LPTY and PPEY domains are required for full activity of AMOT. Unlike AMOT-p130, due to the lack of these motifs, AMOT-p80 fails to bind with YAP1. Thereby, it is AMOT-p130, instead of AMOT-p80, that promotes YAP1 phosphorylation and inhibits YAP1 transcriptional activity [8]. In addition, AMOT undergoes proteasomal degradation. Three members of Nedd4 (neural-precursor-cell-expressed developmentally down-regulated)-like ubiquitin E3 ligases, Nedd4, Nedd4-2 and Itch, mediate poly-ubiquitination of AMOT-p130 [8]. Nedd4-like ubiquitin E3 ligases competes with YAP1 to bind AMOT-p130 and subsequently targets AMOT-p130 for ubiquitin-dependent degradation [8]. Ubiquitin ligase atrophin-1 interacting protein 4 (AIP4) is a member of the Nedd4 family. AMOT-p130 is ubiquitinated by AIP4 at residue Lys-481 [64]. AMOT-p130 activates AIP4, resulting in the catalysis of ubiquitination of AIP4, AMOT-p130 and YAP1, meaning that AMOT-p130 works interdependently with AIP4 to reduce the stability of YAP1 and inhibit YAP1-dependent transcription as well as cell growth [64]. Hence, AMOT-p130 and AIP4 cooperatively mediate anti-growth partly through the inhibition of YAP1 [64]. AMOTs could also serve as direct activators of LATS2. AMOTL1, AMOTL2 and AMOT-p130 bind with LATS2 and YAP1 to promote LATS2 phosphorylating YAP1 [65]. Post-translational modifications of AMOT family proteins are critical for their context-dependent functions during carcinogenesis. The N-terminal domain of AMOT contains a consensus motif (HxRxxS) for phosphorylation by LATS1/2 [18]. The phosphorylation of AMOT at Serine 176 shifts localization of this complex to the plasma membrane, where it correlates with the tight-junction proteins Pals1/PATJ and E-cadherin. Therefore, phosphorylation of AMOT S175 is a critical post-translational modification that suppresses YAP1’s ability of promoting cell proliferation and tumorigenesis [47]. The phosphorylation of AMOT-p130 by LATS is a key feature that enables it to inhibit YAP1-dependent signaling and cell growth [66]. Phosphorylation of AMOT by LATS1/2 inhibits F-actin binding, cell migration and angiogenesis [16]. AMOT S175 is crucial for the actin-binding activity. Wild-type and non-phosphorylated AMOT (AMOT-S175A) interact with actin filaments, whereas phospho-mimic AMOT (AMOT-S175D) fails to be localized with actin [67]. S175A promotes cell proliferation, while AMOT and S175D inhibits it [67]. Motin family members are required for YAP1 cytoplasmic relocalization in response to the actin cytoskeleton perturbation [68]. F-actin and YAP1 compete to bind with AMOT-p130, accounting for how F-actin inhibits AMOT-p130-mediated cytoplasmic retention of YAP1 [68]. Furthermore, LATS synergizes with F-actin perturbations by phosphorylating free AMOT-p130 to keep it from interacting with F-actin [68]. Mutants’ defective of AMOTs for F-actin binding shows an enhanced ability to retain YAP1 in the cytoplasm. LATS2-phosphorylated AMOT-p130 inhibits the association of AMOT-p130 with F-actin, and AMOT-p130 binding to F-actin inhibited LATS phosphorylation. In short, F-actin and YAP1 competes to bind with AMOT-p130 [68]. Down-regulation of AMOTL2 promotes epithelial-mesenchymal transition (EMT), which was also observed when YAP1 is overexpressed [69].

### Oncogenic functions of AMOTs

In some specific cancer types, oncogenic functions of AMOT have also been revealed. RNF146 (an E3 ubiquitin ligase that recognizes ADP-ribosylated substrates) and tankyrase stabilize the Crumbs complex through downregulation of AMOT proteins at the apical membrane [70]. In breast cancer, AMOT was observed to be highly expressed in cancer tissues comparing with adjacent non-cancerous tissues. Decreased AMOT inhibited cell proliferation and invasion. In addition, the expression of YAP1 and LATS1 was suppressed, especially the expression of YAP1, which was significantly decreased [71]. In renal cell carcinoma (RCC), AMOT maintained nuclear localization of YAP so as to increase cell proliferation [72]. Atypical protein kinase C1 (PKC1) was suggested to be an oncogene in lung and ovarian cancer. Knockdown PKC1 enhanced the binding between YAP1 and AMOT, which retained YAP1 in the cytoplasm. PKC1 directly phosphorylated AMOT at Thr750, whose phosphorylation decreases YAP1 binding. PKC1-AMOT-YAP1 signaling axis enhanced the growth of ovarian serous carcinoma tumor [73]. AMOT-p80 functions as a tumor promoter by enhancing PCa cell proliferation. AMOT-p80 promotes nuclear localization of YAP1 through the Hippo pathway, which resulting in an overexpression of YAP1 targeting protein BMP4 [74]. Oncogenic functions of AMOT-p130 have also been justified. Yi et al. [75] suggested that AMOT-p130 was required for YAP1-mediated hepatic epithelial cell proliferation and tumorigenesis. Mice with a liver-specific AMOT knockout presented reduced hepatic ‘oval cell’ proliferation and tumorigenesis in response to toxin-induced injury [75]. These researchers also revealed that AMOT-p130 interacts with YAP1 in both cytoplasm and nucleus. In the cytoplasm, AMOT-p130 prevented the phosphorylation of YAP1 by blocking the access of the WW domains to the kinase LATS1 [75]. In the nucleus, AMOT-p130 interacted with the transcriptional complex containing YAP1 and TEADs to induce the expression of downstream targets [75]. Based on the facts above, AMOT promoted YAP1 nuclear translocation and acted as a transcriptional co-factor of the YAP1-TEAD complex to facilitate proliferation of biliary epithelial cells and cancer development of the liver [75]. In glioblastoma, phosphorylation of AMOTL2 by the mTORC2 kinase enhances YAP1 signaling, leading to cancer growth and invasiveness [76].

## AMOTs involved in other molecular mechanisms

In hemangioendothelioma invasion, AMOT promotes endothelial invasion by both stimulating invasion and stabilizing established tubes [77]. In human breast tumor tissues, AMOT was reported to be highly expressed, which was related to angiogenesis [78]. There was evidence that a DNA vaccine targeting AMOT overcame immune tolerance and hampered the progression of incipient tumors [79]. In addition, angiogenesis and tumor growth were inhibited in vivo with targeting AMOT DNA vaccination [80]. Silencing of AMOT prevented the activation and angiogenic effects [81]. In clear cell renal cell carcinoma (ccRCC), AMOT transcripts were associated with poor differentiation, venous invasion, which made it an independent prognostic factor for survival of ccRCC patients [82]. Overexpression of AMOT has also been detected in sinonasal inverted papilloma [83]. In head and neck squamous cell carcinoma (HNSCC), through regulating AMOT-p80 expression, different cell behaviors are induced. For instance, its high expression spured cells proliferate and migrate while its low level induces invasion or metastasis [84]. In prostate cancer, AMOT-p80 functioned as a component of the Cadl1 protein complex, which played a role in cell migration [85]. In colorectal cancer, upregulation of AMOT has been observed. Overexpressed AMOT promoted cell proliferation, cell invasion and migration, as well as apoptotic resistance to 5-fluorouracil. Moreover, AMOT abundance also led to the activation of ERK and AKT pathways [86]. In human osteosarcoma cells, elevated AMOT expression facilitated cell growth and migration [87], whereas its silencing decreased cell proliferation, migration and invasion [88]. A similar results was detected in breast cancer accordingly [89]. In human breast and colon cancer patients, AMOTL2 expression was associated with deficiency of tissue architecture. Hypoxic-stress-induced AMOTL2 activation promoted the loss of polarity [90]. AMOTL2 interacting with AKT and negatively regulating AKT have been identified recently [91]. In vivo, Liver-specific depletion of AMOTL2 enlarged mouse liver, which was correlated with the concomitant activation of YAP and AKT [91]. These observations suggested a dual tumor suppressive function of AMOTL2 through targeting both YAP and AKT [91].

## Other functional roles of Motin family

AMOTs have been identified to be essential among viruses as well. Based on previous studies, AMOT-p130 was a host protein required for efficient HIV-1 release, which bound with both NEDD4L and HIV-1 Gag to stimulate HIV-1 release. In addition, expression of either AMOTL1 or AMOTL2 also promoted HIV-1 release and infectivity when AMOT-p130 is absent [92]. Moreover, AMOTL1 has been suggested to play a role in paramyxovirus infection, since AMOTL1 deletion reduced the budding of parainfluenza virus 5 (PIV5) [93]. A raising expression of AMOT was observed among rheumatoid arthritis (RA) patients. However, there was no evidence suggesting a correlation between AMOT level and other clinical variables [94]. In rat models of incipient diabetic nephropathy, inhibition of AMOT reduced glomerular hypertrophy and periodic acid-Schiff positivity. Consequently, the progression of diabetic nephropathy was found to be inhibited [95]. In lung cells, interaction between AMOTL2 and TAZ inhibits surfactant proteins expression. Besides, abundant AMOTL2 inhibits TAZ nuclear distribution, which subsequently decreases the expression of target genes [96]. In mammalian skeletal muscle, AMOTL2 associates with synaptic podosomes in cultured myotubes and it regulates postsynaptic differentiation in muscle cells [97].

## Conclusion

Taken together, the functional roles of AMOT-p80, AMOT-p130, AMOTL1, and AMOTL2 in different cancer types are controversial and they highly depend on cell context. In some cancer types, AMOTs promote cell proliferation and invasion, including breast cancer, renal cell carcinoma, colon cancer, prostate cancer, and cervical cancer. However in lung cancer and glioblastoma, AMOTs inhibit tumor growth. In addition, the functional roles of AMOTs in Hippo-YAP1 signaling are still elusive. AMOT promotes either YAP1 nuclear localization or cytoplasmic retention in different cancer types. Moreover, the protein expression of AMOT members varies depending on the cell density. For examples, AMOT protein expression is upregulated in dense-cell condition. In different cancer types, AMOTs play either oncogenic or tumor suppressive role. The mechanisms may rely on the following issues. Firstly, there are three family members of AMOT family. Their expression patterns are distinct, indicating the functional roles of AMOTs might be different. Secondly, the functional roles of different AMOT members are contradictory in different cancer types. In this review, the knowledge about AMOTs is summarized and hints are offered for further investigation.

Therefore, more studies are required to elucidate the controversial function of AMOTs in carcinogenesis. Based on our summary above, several issues need to be addressed in the future study. Firstly, which AMOT family member are predominantly expressed in some specific cancer cells or tissues? Secondly, what is the real function of AMOTs and what are the detailed molecular mechanisms determining the oncogenic or tumor-suppressive function of AMOTs? Thirdly, what are the crucial upstream regulators and downstream effectors of AMOTs in tumorigenesis? Elucidation of AMOTs’ real function and mechanisms may lead to novel therapeutic strategies and promote anti-cancer drug development.

## Abbreviations

AMOT: Angiomotin
AMOTL1: Angiomotin-like 1
AMOTL2: Angiomotin-like 2
BAR domain: Bin/Amphiphysin/Rvs domain
TE: trophectoderm
ICM: inner cell mass
Rock: Rho-associated kinase
KD: knockdown
AVE: anterior visceral endoderm
VE: visceral endoderm
TIE: tyrosine kinase
Patj: Pals1-associated TJ protein
Pals1: Lin Seven 1
Syx: synectin-binding GEF
DMSCs: dermal mesenchymal stem cells
TFPI-1: tissue factor pathway inhibitor-1
ZO-1: zonula occludens protein 1
TJs: tight junctions
HECW2: HECT, C2 and WW domain containing E3 ubiquitin protein ligase 2
MDCK: Madin–Darby canine kidney
Rich1: RhoGAP interacting with Cdc42-interacting protein four homologues protein 1
MUPP1: multi-PDZ-domain protein 1
Malat1: metastasis-associated lung adenocarcinoma transcript 1
SRSF1: serine/arginine-rich splicing factor 1
EGFR: endothelial growth factor receptor
RNF146: an E3 ubiquitin ligase that recognizes ADP-ribosylated substrates
RCC: renal cell carcinoma
PKC1: protein kinase C1
Nedd4: neural-precursor-cell-expressed developmentally down-regulated
AIP4: atrophin-1 interacting protein 4
EMT: epithelial-mesenchymal transition
HNSCC: head and neck squamous cell carcinoma
PIV5: parainfluenza virus 5
EDTB: endosomal integral membrane protein endotubin
RA: rheumatoid arthritis
GC: gastric cancer
TCGA: The Cancer Genome Atlas
